# PKM2, a Central Point of Regulation in Cancer Metabolism

**DOI:** 10.1155/2013/242513

**Published:** 2013-02-14

**Authors:** Nicholas Wong, Jason De Melo, Damu Tang

**Affiliations:** ^1^Division of Nephrology, Department of Medicine, McMaster University, Hamilton, ON, Canada L8S 4L8; ^2^Division of Urology, Department of Surgery, McMaster University, Hamilton, ON, Canada L8S 4L8; ^3^Father Sean O'Sullivan Research Centre, St. Joseph's Healthcare Hamilton, Hamilton, ON, Canada L8N 4A6; ^4^The Hamilton Center for Kidney Research, St. Joseph's Healthcare Hamilton, Hamilton, ON, Canada L8N 4A6

## Abstract

Aerobic glycolysis is the dominant metabolic pathway utilized by cancer cells, owing to its ability to divert glucose metabolites from ATP production towards the synthesis of cellular building blocks (nucleotides, amino acids, and lipids) to meet the demands of proliferation. The M2 isoform of pyruvate kinase (PKM2) catalyzes the final and also a rate-limiting reaction in the glycolytic pathway. In the PK family, PKM2 is subjected to a complex regulation by both oncogenes and tumour suppressors, which allows for a fine-tone regulation of PKM2 activity. The less active form of PKM2 drives glucose through the route of aerobic glycolysis, while active PKM2 directs glucose towards oxidative metabolism. Additionally, PKM2 possesses protein tyrosine kinase activity and plays a role in modulating gene expression and thereby contributing to tumorigenesis. We will discuss our current understanding of PKM2's regulation and its many contributions to tumorigenesis.

## 1. Introduction

**Figure 1:**
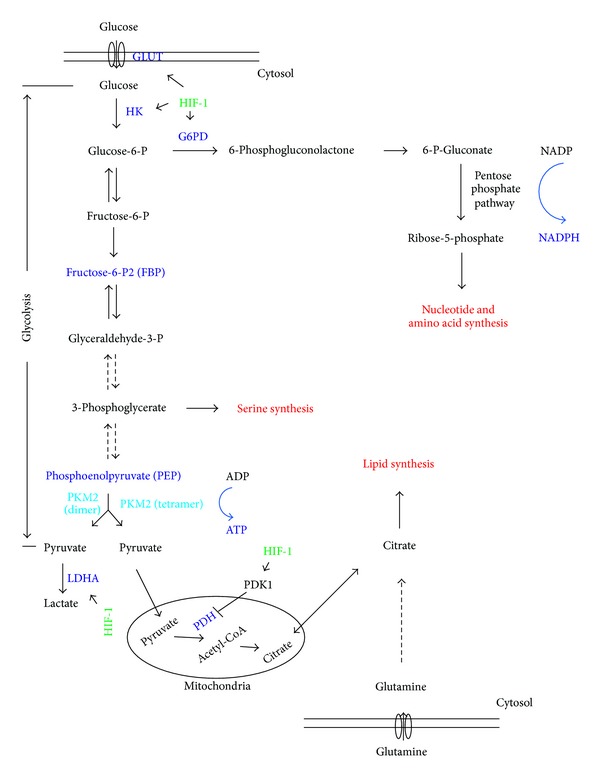
Schematic illustrating the cancer utilization of the metabolic pathways. Pyruvate kinase catalyzes the last step of glycolysis by converting PEP and ADP to pyruvate and ATP, respectively. PKM2 dimers and tetramers possess low and high levels of Pyruvate kinase activity, respectively. With reduced enzymatic activity, PKM2 dimer drives aerobic glycolysis, which allows the intermediate metabolites to be used for the synthesis of nucleotides, amino acids, and lipids and the production of reduced NADPH (see the pentose phosphate pathway). HIF-1 upregulates the indicated proteins. GLUT: glucose transporter, HK: hexokinase, G6PD: glucose-6-phosphate dehydrogenase, HIF-1: hypoxia-inducible factor 1, LDHA: lactate dehydrogenase A, PDK1: pyruvate dehydrogenase kinase isoenzyme 1, and PDH: pyruvate dehydrogenase.

**Figure 2:**
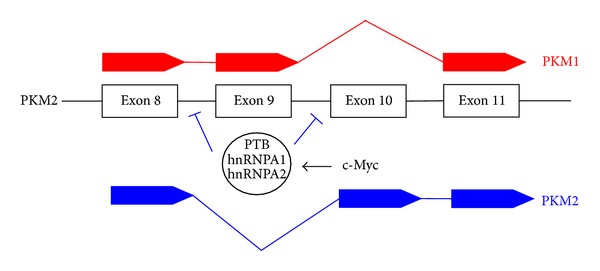
Schematic illustration of alternative splicing of PKM1 and PKM2. The proportion of the *PKM2* gene is shown. c-Myc upregulates the indicated complex which inhibits the splicing for exon 9, resulting in its exclusion in PKM2. PTB: polypyrimidine track binding protein; hnRNPA1 and hnRNPA2: heterogeneous nuclear ribonucleoprotein 1 and 2.

Metabolism lies in the heart of cell biology. Understanding how cancer cells cope with metabolic needs for their unique biology has been a focus of cancer research for many years. It began with the landmark observation reported more than 80 years ago by Otto Warburg that cancer cells consumed more glucose and produced a large amount of lactate even in a well-oxygenized environment, a process known as aerobic glycolysis or the Warburg effect [1, 2]. While normal differentiated cells maximize ATP production by mitochondrial oxidative phosphorylation of glucose under normoxic conditions, cancer cells generate much less ATP from glucose by aerobic glycolysis. Despite being less efficient in ATP production, glycolysis is a much more rapid process [3, 4]. Cancers commonly deregulate pathways that enhance glycolysis, including activation of the PI3 K-ATK-mTOR pathway and upregulation of HIF-1 and c-Myc [5, 6]. The increase in aerobic glycolysis together with its dynamic process in cancer cells enables glycolytic intermediates to be redirected for the biosynthesis of cellular building blocks (nucleotides, amino acids, and lipids) while also producing ATP. Therefore, the Warburg effect/aerobic glycolysis meets the demands of cancer and proliferating cells for macromolecular synthesis and energy production [7, 8]. As a result, cancer cells display enhanced glucose uptake and produce higher levels of lactate [1, 2]. The Warburg effect was explored for the common clinical detection of tumors by fluorodeoxyglucose (2-deoxy-2-(^18^F)fluoro-D-glucose) positron emission tomography (FDG-PET) [7]. In the glycolytic process, pyruvate kinase (PK) catalyzes the last reaction, transfer of a high-energy phosphate group from phosphoenolpyruvate (PEP) to ADP, producing ATP and pyruvate [9]. Pyruvate is then either reduced to lactate by lactate dehydrogenase (LDH) in the cytosol or enters the mitochondria to produce ATP through the tricarboxylic acid (TCA) cycle (Figure 1). Along the glycolysis pathway, intermediate metabolites can be channeled to synthesize amino acids, nucleotides, and lipids (Figure 1) if the rate of flux through the pathway is controlled. PK is an ideal candidate for this control [10] because (1) PK catalyzes the last reaction of the pathway (Figure 1) and (2) the reaction is essentially irreversible (Figure 1) [9, 11]. Therefore, lowering PK activity is expected to produce less pyruvate (Figure 1) or prevent complete conversion of glucose to pyruvate (1 molecule of glucose to 2 molecules of pyruvate). This enables the upstream glycolytic intermediates to accumulate and thus contribute to the shift of metabolism towards the anabolic phase for amino acids, nucleotides, and lipid production (Figure 1). Cancer cells explore this logic by predominantly using PKM2, an isoform of PK, as its activity can be dynamically regulated between the less active PKM2 dimer and the highly active PKM2 tetramer [12].

PK consists of four isoforms: the L (PKL) and R (PKR) isoforms encoded by the *PKLR *(1q22) gene and the M1 (PKM1) and M2 (PKM2) isoforms encoded by the *PKM2 *(15q23) gene. The *PKLR *gene is regulated by tissue-specific promoters. The full-length PKR isoform is expressed in red blood cells while the PKL isoform missing exon 1 is detected in liver and kidney [5, 13, 14]. PKM1 and PKM2 are produced from the *PKM2 *gene by alternative splicing [15]. The highly active PKM1 is expressed in tissues that consistently need high levels of energy, like skeletal muscle, heart, and brain [5, 10]. PKM2 is expressed in most cells except adult muscle, brain, and liver [12, 16, 17] and is the predominant PK in proliferating and cancer cells [18]. While PKL, PKR, and PKM1 form stable tetramers (the active form of PK), PKM2 exists as both dimers and tetramers [18, 19]. The PKM2 dimer has a higher *K*_*m*_ towards PEP than the tetramer and thus is less active in converting PEP to ATP and pyruvate [19, 20]. While tetrameric PKM2 favors ATP production through the TCA cycle, dimeric PKM2 plays a critical role in aerobic glycolysis (Figure 1) [19]. Therefore, the dynamic equilibrium between dimer and tetramer PKM2 allows proliferating cells to regulate their needs for anabolic and catabolic metabolism. This not only explains why cancer cells predominantly express PKM2 but also reveals the existence of mechanisms that regulate this dynamic equilibrium. To ensure PKM2 expression, cancer cells also develop mechanisms for alternative splicing to produce PKM2 rather than PKM1. These mechanisms are regulated by oncogenes and tumor suppressors [21–25]. Surprisingly, dimeric PKM2 has additional functions in regulating gene expression in the nucleus [26].

## 2. PKM2 Contributes to Tumorigenesis

A large body of evidence supports the notion that cancers predominantly express PKM2 [14]. Immunohistochemical analysis revealed that PKM2 is commonly expressed in colon cancer [12], renal cell carcinoma (RCC) [27], and lung cancer [28]. PKM2 has been suggested to be a marker for RCC [29, 30] and testicular cancer [31]. Elevation of serum PKM2 levels was reported in patients with colon cancer [32], breast cancer [33], urological tumors [34], lung carcinoma, cervical cancer, and gastrointestinal tumor [18]. PKM2 was detected in the feces of patients with gastric and colorectal cancers [35]. Recently, mass spectrometry has demonstrated increases in PKM2, and the predominant presence of PKM2 was confirmed in RCC, bladder carcinoma, hepatocellular carcinoma, colorectal cancer, lung carcinoma, and follicular thyroid adenoma [16].

PKM2 expression correlates with tumorigenesis. High levels of PKM2 associate with poor prognosis for patients with signet ring cell gastric cancer [36]. A unique pattern of four expressed genes, including PKM2, was reported to predict outcomes for mesothelioma patients undergoing surgery [37]. Events that negatively impact tumorigenesis can also reduce PKM2 function. Vitamins K3 and K5 inhibit tumorigenesis along with potently inhibiting PKM2 activity [38]. Butyrate displays anticolon cancer effects along with the inhibition of PKM2 expression in neoplastic but not nontumor colon tissues [39]. Shikonin, a derivative of a Chinese herb with antitumor activities, induces necrosis and inhibits PKM2 expression in cancer cell lines [40]. A reverse correlation was observed between antitumor microRNA-326 and PKM2 in glioma [41]. Finally, the Spry2 tumor suppressor was reported to inhibit hepatocarcinogenesis via the MAPK and PKM2 pathways [42].

 Furthermore, PKM2 possesses activities that directly promote tumorigenesis. Overexpression of PKM2 upregulates Bcl-xL in gastric cancer and promotes the proliferation and migration of colon cancer cells [43, 44]. Knockdown of PKM2 using specific siRNA inhibited cancer cell's proliferation and invasion in vitro and the formation of xenograft tumors in vivo [41, 45].

## 3. PKM2 Promotes Tumorigenesis via Regulating the Warburg Effect

The needs of energy production (ATP) and synthesis of cellular building blocks for proliferating cancer cells dictate the shift from oxidative to glycolytic metabolism even under normoxic conditions, the Warburg effect or aerobic glycolysis [2, 7, 8]. Under hypoxic conditions, cells metabolize glucose by anaerobic glycolysis, a process that is regulated by two master transcription factors, hypoxia-inducible factor (HIFs), and c-Myc [46]. Both transcriptional factors are also critical for aerobic glycolysis in cancer cells. Consistent with PKM2 being essential for aerobic glycolysis, a relationship exists among HIF-1, c-Myc, and PKM2. We will discuss the current understanding of these relationships.

### 3.1. Positive Feedback Regulation between PKM2 and HIF-1

It was first demonstrated by Christofk and colleagues in 2008 that knockdown of PKM2 in a panel of cancer cell lines decreased the rate of glycolysis and proliferation. Introducing PKM2 but not PKM1 to the knockdown cells not only enhanced glycolysis but also increased the ability to form xenograft tumors [12]. This research elegantly revealed that PKM2 is important and that the level of PK activity is essential, as the defects in PKM2 knockdown cells in supporting tumorigenesis could not be corrected by overexpression of the more active isoform PKM1. Furthermore, in comparison to PKM1 rescued cells, reintroducing PKM2 into knockdown cells rescued the deficiency of cell proliferation under hypoxic conditions.

This investigation also suggests that PKM2 may contribute to the adaptive response (hypoxia response) of cells to hypoxia, which is specifically relevant to tumorigenesis as solid cancers consistently face hypoxia intratumorally. It is thus a typical characteristic that cancers consistently execute hypoxia response. In the heart of this response lies the master transcription factor, hypoxia-inducible factor 1 (HIF-1) [47]. HIF-1 is a heterodimeric transcription factor, consisting of HIF-1*α* and HIF-1*β*. The *β* subunit is constitutively expressed, while the *α* subunit is directly regulated by oxygen (O_2_) levels [48, 49]. Under normoxic conditions, HIF-1*α* is hydroxylated at prolines (P) 402 and 564 by three prolyl hydroxylase domain proteins (PHD1-3) in the presence of oxygen, *α*-ketoglutarate, iron, and ascorbate [50]. This results in the ubiquitination of prolyl-hydroxylated HIF-1*α* by the von Hippel-Lindau (VHL) tumor suppressor and the subsequent degradation of HIF-1*α* [51, 52]. Under hypoxic conditions, HIF-1*α* is stabilized as a result of inhibiting prolyl hydroxylation, allowing HIF-1*α* to dimerize with HIF-1*β* in the nucleus. This leads to transcription of a set of genes to cope with reduced O_2_ availability [53–55]. These target genes include those responsible for promoting glycolysis [56]. HIF-1 transactivates the glucose transporters GLUT1 and GLUT3, hexokinase (the first kinase in the glycolysis pathway), lactate dehydrogenase A (LDHA), and pyruvate dehydrogenase kinase 1 which phosphorylates and inhibits pyruvate dehydrogenase (PDH) [57] (Figure 1). Consistent with the Warburg effect's association with synthesis of cellular building blocks, HIF-1 also transactivates glucose-6-phosphate dehydrogenase (G6PD) to channel glucose-6-P into the pentose phosphate shunt for nucleotide and amino acid synthesis (Figure 1) [56]. Therefore, the collective actions of HIF-1 transcription activity seem to shift cells from oxidative metabolism to glycolysis (Figure 1). In line with these observations, PKM2 shares an intimate connection with HIF-1. The first intron of the *PKM2* gene contains the functional hypoxia-response element (HRE), thus also making it a target of HIF-1 [21].

PKM2 also possesses a positive feedback regulation towards HIF-1. PKM2 interacts with HIF-1*α*, a process that requires the prolyl hydroxylase 3 (PHD3). PHD3 binds to and causes hydroxylation of PKM2 at P303/408. This association and hydroxylation induces PKM2 to interact with HIF-1*α*, which plays a role in HIF-1-mediated transactivation of target genes including the *LDHA*, *PDK1*, and *VEGFA* (encoding the vascular endothelial growth factor) genes [21]. Additionally, PKM2 binds to p300 and enhances its recruitment to the HRE sites of HIF-1 target genes. Taken together, PKM2 functions as a HIF-1 coactivator by enhancing the Warburg effect in cancers [21, 22].

The regulation between HIF-1 and PKM2 also occurs under normoxic conditions, by changes in other signalling events which act to stabilize HIF-1*α* in cancer cells. HIF-1 is stabilized by mTOR and induced for degradation by VHL. Activation of mTOR is inhibited by tumor suppressors TSC1/TSC2 and facilitated by the PI3 K-AKT pathway [57]. Consistent with this knowledge, abnormal activation of the PI3 K-AKT-mTOR pathway and loss of function of tumor suppressors VHL, TSC1/2, and PTEN have been demonstrated to stabilize HIF-1*α* [57, 58]. Activation of mTOR by downregulation of TSC1/2 and PTEN induced PKM2 expression via stabilization of HIF-1*α* [59]. PKM2 makes essential contributions to mTOR-mediated aerobic glycolysis, as knockdown of PKM2 reduced glucose consumption and lactate production in cells with elevated mTOR activation. Furthermore, downregulation of PKM2 also suppressed mTOR-mediated tumorigenesis [59].

### 3.2. Positive Feedback Regulation between PKM2 and c-Myc

The *PKM2* gene produces both M1 and M2 isoforms through alternative splicing. The difference between these is the inclusion of exon 9 and exclusion of exon 10 for PKM1 and vice versa for PKM2 (Figure 2) [5, 15]. This mutually exclusive pattern of splicing is mediated by members of the heterogeneous nuclear ribonucleoprotein (hnRNP) family, hnRNPA1, hnRNPA2, and hnRNP1/PTB (polypyrimidine track binding protein) [23, 60]. Binding of these proteins to the DNA sequence flanking exon 9 prevents its inclusion, resulting in the inclusion of exon 10 [23, 60, 61]. In order to achieve predominant expression of the M2 isoform, cancer cells have a strategy to preferentially splice the M2 isoform over M1 through c-Myc-mediated upregulation of hnRNPA1, hnRNPA2, and PTB (Figure 2) [23, 61]. This finding is supported by the discovery that cells with high levels of c-Myc activity also demonstrated high PKM2/PKM1 ratios [23, 62]. These observations are well in line with a large body of evidence indicating that c-Myc stimulates glycolysis and is required to coordinate with HIF-1 to regulate the cellular response to hypoxia [24, 46]. Thus, evidence suggests that PKM2 plays a role in c-Myc-mediated cancer metabolism and in c-Myc's communication with HIF-1. Adding to this attractive possibility is a recent demonstration that PKM2 also upregulates c-Myc transcription [63, 64], suggesting another positive feedback loop involving PKM2 in regulating the Warburg effect. Taken together, PKM2 is an integrated piece in the network of glycolysis regulation together with HIF-1 and c-Myc. The importance of hnRNPA1, hnRNPA2, and PTB in splicing PKM2 has also been explored by tumour suppression activity. The microRNAs mir-124, mir-137, and mir-340 inhibit colorectal cancer growth by repressing the expression of these hnRNAs favouring PKM1 splicing, thereby inhibiting aerobic glycolysis or the Warburg effect [65].

## 4. Regulation of PKM2 in the Warburg Effect during Tumorigenesis

Cancers have developed a complex regulation of PKM2 to meet the needs for energy and synthesis of nucleotides, amino acids, and lipids. These mechanisms center on regulating PKM2's expression, allosteric regulation, and modifications. The latter two mechanisms directly or indirectly affect PKM2 activity through physical interaction and by regulating the PKM2 dimer-tetramer dynamic.

### 4.1. Transcription Regulation

In addition to the above discussion of HIF-1 and c-Myc-mediated transcription and splicing of PKM2, transcription of the *PKM2* gene is also regulated by the SP1 and SP3 transcription factors [5, 22, 66]. The network of PI3 K-AKT-mTOR (mammalian target of rapamycin) plays a critical role in cell metabolism, proliferation, and survival and is one of the most frequently activated pathways in cancer owing to the activation of kinases and the inactivation of tumor suppressors, TSC1/2 (tuberous sclerosis 1/2) and PTEN [67]. Nutrient status is well known to modulate mTOR activation [68]. Under normoxic conditions, mTOR activity induces PKM2 expression through the combination of HIF-1*α* and c-Myc [59, 69]. Inhibition of mTOR has been found to reduce glycolysis and PKM2 expression [70]. Elevation in PTEN function reduces glucose uptake and the Warburg effect and inhibits PKM2 expression [25]. In a feedback manner, PKM2 is able to sustain mTOR activation in serine-depleted medium by enhancing endogenous serine synthesis [71]. Taken together, evidence supports that the upregulation of PKM2 plays an important role in the mTOR-mediated Warburg effect in tumors.

### 4.2. Regulation of the Dimer-Tetramer Dynamics

Tumor cells express high levels of dimer PKM2 [14, 32]. Among the four PK isoforms, PKM2 is the only one to be allosterically regulated between a less active dimer and an active tetramer [18, 19]. These different forms of PKM2 regulate glucose metabolism through either the TCA cycle or glycolysis. Accumulating evidence supports the concept that the less active PKM2 dimer drives aerobic glycolysis, while the active PKM2 tetramer produces pyruvate for oxidative phosphorylation (Figure 1) [12, 72–74]. PKM2 is regulated by fructose-1,6-biphosphate (FBP), an upstream intermediate of glycolysis which when bound to PKM2 activates tetramerization through high affinity association [75–77]. Binding of tyrosine-phosphorylated peptides dissociates FBP from the PKM2 tetramer, resulting in conversion to the PKM2 dimer [72]. The less active PKM2 dimer is critical in mediating aerobic glycolysis in tumor cells based on high levels of lactate production and lower oxygen consumption [72]. Disrupting the binding of the phosphotyrosine peptide in a PKM2 mutant (M2KE) increased PKM2 kinase activity, which was associated with reduction in lactate production and elevation of oxygen consumption [72]. In supporting the low levels of cellular pyruvate kinase activity being critical for aerobic glycolysis, replacing PKM2 with PKM1 led to an increase in cellular pyruvate kinase activity, decreasing lactate production and elevating oxygen consumption [12, 74].

Collectively, evidence supports that the PKM2 dimer is critical in mediating aerobic glycolysis. In addition to the above mechanism regulating PKM2 activity, PKM2 was also controlled by tyrosine phosphorylation [73]. It was observed in 1988 that PKM2 was tyrosine-phosphorylated in v-Src-transformed chicken embryo cells. This phosphorylation reduced the affinity of PKM2 towards its substrate phosphoenolpyruvate (PEP) [78]. In vitro, v-Src was able to directly phosphorylate PKM2 [78]. Although this investigation suggested that v-Src phosphorylated PKM2, the sites of phosphorylation remain unknown. Recent development demonstrated that PKM2 was phosphorylated at several tyrosine residues, including Y105, by fibroblast growth factor receptor type 1 (FGFR1) [73]. Phosphorylation at Y105 causes FBP to dissociate from the PKM2 tetramer, which results in PKM2 dimers and promotes the Warburg effect based on the production of lactate [73]. Conversely, abolishing Y105 phosphorylation by substitution with phenylalanine (Y105F) elevated the kinase activity, resulting in decreased lactate production and increased oxygen consumption [73]. Taken together, evidence demonstrates that phosphorylation at Y105 plays a role in the conversion of PKM2 tetramers to dimers.

More importantly, regulation of PKM2 dimer and tetramer conversion is critical for tumorigenesis. While the less active PKM2 dimer enhances xenograft tumor formation, enforced formation of active PKM2 (KE and Y105F mutations) and replacing PKM2 with PKM1 inhibited the formation of xenograft tumors [72, 73, 79]. In line with this concept, the conversion between dimer and tetramer PKM2 is also used in tumour suppression to inhibit tumorigenesis. The death-associated protein kinase (DAPK) tumor suppressor activates PKM2 by stabilizing the PKM2 tetramer via a direct association. This reduces cancer metabolism or the Warburg effect, which may be one aspect of DAPK-mediated tumor suppression [80, 81].

In line with these observations, several small molecule PKM2 activators have been identified. Among them, DASA-58 (the substituted N, N′-diarylsulfonamide NCGC00185916) and TEPP-46 (the thieno-[3,2-b]pyrrole [3,2-d]pyridazinone NCGC00186528) activate PKM2 by inducing PKM2 tetramerization. Unlike FBP-induced activation, the tetramer induced by these compounds is resistant to tyrosine-phosphorylated peptide-mediated conversion to the PKM2 dimer. This suggests that FBP and these small molecule activators bind PKM2 at distinct sites, but, importantly, all inhibit tumorigenesis [74, 82, 83]. Additionally, a new set of chemical platform bases, the quinolone sulfonamide-based PKM2 activators, have recently been reported. Similar to DASA-58 and TEPP-46, these activators also stabilize the PKM2 tetramer via binding to a pocket distinct from FBP binding and thus prevent the PKM2 tetramer from tyrosine-phosphorylated peptide-mediated disruption. Quinolone sulfonamide-based PKM2 activators reduce carbon flow towards the serine biosynthetic pathway, rendering cells to serine auxotrophy [84].

### 4.3. Factors Affecting PKM2 Activity via Physical Association

In addition to the above two small molecule PKM2 activators, a third activator was recently reported by the same research group based on modifications to one of their previous compounds [85]. The mechanism underlying this activation remains to be defined. A series of PKM2 activators (1-(sulfonyl)-5-(arylsulfonyl)indoline) were also reported very recently [86]. In contrast to these, potent small molecule PKM2 inhibitors which may in part induce cell death by inhibiting PKM2 activity have also been developed [87]. Furthermore, the pyruvate kinase activity of PKM2 can be inhibited by association with several distinct proteins. While the nuclear promyelocytic leukemia (PML) protein functions as a tumor suppressor, cytosolic PML was reported to specifically inhibit tetrameric but not dimeric PKM2 activity, thereby contributing to the Warburg effect [88]. Prolactin signal promotes cell proliferation by inducing its receptor to associate with PKM2, leading to PKM2 activity reduction [89]. The MUC1-C oncoprotein was reported to promote breast cancer tumorigenesis in part via inhibiting PKM2 activity. Although interaction of MUC1-C Cys3 with PKM2 C-domain Cys474 results in activation of PKM2, oncogenic signals from EGFR (epidermal growth factor receptor) can alter the association of MUC1-C and PKM2, thereby leading to inhibition of PKM2 activity [90]. EGFR phosphorylates MUC1-C at tyrosine 46, causing MUC1-C to interact with PKM2 at Lys433. This association inhibits tetrameric PKM2 activity and thereby increases aerobic glycolysis along with glucose uptake [90]. PKM2 was also found to interact with human papillomavirus 16 (HPV16) protein E7, which may contribute to HPV16-induced cervical cancer [91]. A potential therapeutic protein TEM8-Fc, consisting of a portion of the tumor endothelial marker 8 (TEM8) and the Fc domain of human IgG1, was found to associate with PKM2 [92]. Whether this interaction contributed to TEM8-Fc-associated tumor suppression was not clear [92]. Consistent with the knowledge that PKM2 plays a critical role in regulating aerobic glycolysis and biosynthesis for cellular building blocks, PKM2 is activated by serine but inhibited by alanine and phenylalanine when bound to these amino acids [93].

### 4.4. Posttranslational Modifications of PKM2

A reduction in activity was reported by acetylation of PKM2 at lysine (K) 305 in response to high levels of glucose. This modification reduces PKM2 activity and its affinity towards the PEP substrate, resulting in PKM2 degradation via chaperone-mediated autophagy [94]. As a result, acetylation enhances cell proliferation by increasing the availability of glycolytic intermediates for anabolic synthesis [94, 95].

PKM2 also plays a role in cell survival to oxidative stress. Acute increases in intracellular levels of ROS (reactive oxygen species) induce oxidation of PKM2 at Cys358. This reduces PKM2 activity, which allows the accumulation of glucose-6-phosphate and thus shifts glucose flux through the pentose phosphate pathway (PPP) to generate reduced NADPH (Figure 1). As PPP is the major pathway of generating reduced NADPH, oxidation-mediated inhibition of PKM2 is therefore a mechanism of detoxification during oxidative stress. Consistent with this notion, substitution of C358 with S358 to produce oxidation-resistant mutants sensitized cells to oxidative stress and inhibited xenograft tumor formation [96]. A similar antioxidative stress function of PKM2 is also mediated through binding to CD44, a major cell adhesion molecule. Cancer stem cells are known to be CD44 positive, so this interaction is consistent with CD44 promoting cancer progression, metastasis, and chemoresistance [97, 98]. CD44's tumorigenic function is in part also attributable to its association with EGFR [99]. Consistent with these observations, PKM2 was reported to bind CD44, resulting in receptor tyrosine kinase-mediated phosphorylation of PKM2 and inhibition of PKM2 activity. This enhanced glucose flux through the PPP pathway to generate reduced NADPH and counteract oxidative stresses through detoxification [61, 100].

## 5. The Nuclear Function of PKM2

**Figure 3:**
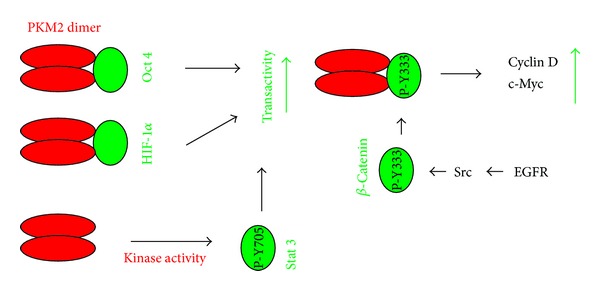
Diagram showing the nuclear function of PKM2. PKM2 dimers in the nucleus bind to Oct 4 and HIF-1*α* and enhance their transcription activity; EGFR signal activates Src tyrosine kinase, which phosphorylates *β*-catenin at tyrosine (Y) 333 (P-Y333). PKM2 binds Y333-phosphorylated *β*-catenin, contributing to *β*-catenin-mediated transcription of cyclin D and c-Myc; PKM2 dimer possesses kinase activity that phosphorylates Stat 3 at Y705, which enhances Stat 3's transcriptional activity.

PKM2 displays intriguing nonglycolytic functions in the nucleus. In addition to its cytoplasmic presence to regulate aerobic glycolysis, PKM2 was also detected in the nucleus in response to interleukin-3 and apoptotic signals [101, 102]. Nuclear PKM2 binds Oct 4 through its C-terminal region (residues 307–531), enhancing Oct-4-mediated transcription [103] (Figure 3). Nuclear PKM2 was also reported to be a coactivator of HIF-1 [21] (Figure 3). EGFR signaling was reported to activate Src tyrosine kinase, which in turn phosphorylates *β*-catenin at Y333. PKM2 binds to tyrosine-phosphorylated *β*-catenin in the nucleus and contributes to *β*-catenin-mediated transactivation of cyclin D and c-Myc, thereby promoting both cell proliferation and tumor progression (Figure 3). This process requires the kinase activity of PKM2 [63, 104]. Since the binding of tyrosine-phosphorylated peptides maintains PKM2 in its dimer status [72], these observations suggest that dimerized PKM2 binds and enhances *β*-catenin function, in which a new kinase activity rather than pyruvate kinase activity might be involved. Indeed, it was very recently reported that the PKM2 dimer contributes to its nuclear function and possesses protein tyrosine kinase activity. Surprisingly, instead of using high-energy ATP, PKM2 uses the high-energy phosphate from PEP as a phosphate donor to phosphorylate its protein substrates [26]. The PKM2 dimer phosphorylates Stat 3 at Y705 in the nucleus and thus enhances Stat 3 transcription activity [26, 105] (Figure 3). Taken together, while tetramer PKM2 is a pyruvate kinase, dimer PKM2 can also act as a protein tyrosine kinase [26].

## 6. Concluding Remarks and Future Perspectives

The last decade has seen a high reemergence of interest in the Warburg effect, the typical cancer cell metabolism that was reported almost 90 years ago. The detailed molecular and genetic knowledge accumulated in the last few decades of extensive cancer research has rapidly advanced our understanding of cancer metabolism. Mutations in several enzymes of the TCA-cycle were discovered, including isocitrate dehydrogenases 1 and 2 (IDH1 and IDH2), succinate dehydrogenase (SDH), and fumarate hydratase (FH) [106–109]. These mutations collectively reduce TCA-cycle-mediated oxidative phosphorylation, resulting in an accumulation of metabolites for the biosynthesis of amino acids, nucleotides, and lipids as well as increases in glucose uptake [110]. The increases in glucose uptake together with aerobic glycolysis yield a robust elevation of lactate production. Although recent development suggests that the by-product of aerobic glycolysis (lactate) contributes to overall tumorigenesis [111, 112], it is also critical for cancer cells to efficiently export lactate to maintain the flux of glycolysis and to prevent cellular acidification [112]. Cancer cells accomplish this task in part by upregulation of the monocarboxylate transporters (MCTs) [112]. Another strategy to reduce the cellular burden of lactate accumulation during aerobic glycolysis may be the prevention of a complete conversion of glucose to lactate (1 glucose for every 2 lactate molecules) by reducing the conversion of PEP to pyruvate. This would allow the glycolytic intermediates to be used for macromolecular synthesis. Therefore, predominantly using the less active PKM2 dimer fits this logic.

Accumulating evidence obtained in the last 10 years demonstrates that PKM2's glycolytic enzyme activity is regulated by oncogenes and tumor suppressors [21–25]. These regulations center on modulation of aerobic glycolysis. Favoring a shift of the dimer-tetramer dynamic towards dimerization is critical for PKM2 to promote the Warburg effect, leading to cell proliferation and tumorigenesis.

Surprisingly, in addition to its glycolytic pyruvate kinase activity in the cytosol, the PKM2 dimer also displays protein tyrosine kinase activity in the nucleus and nuclear PKM2 promotes the transcriptional activities of HIF, *β*-catenin, STAT 3, and Oct 4 [21, 26, 63, 103–105]. This all indirectly contributes to cancer metabolism and other aspects of tumorigenesis. In light of this new development, future research should determine the contributions of the cytosolic versus nuclear PKM2 dimer to aerobic glycolysis.

Effort is currently underway to target PKM2 for cancer therapy, which is part of the current attempt in targeting cancer metabolism. Several small molecule PKM2 inhibitors and activators have been developed [61]. As nearly complete knockdown of PKM2 does not completely inhibit cancer cell proliferation, the utility of PKM2 inhibition in targeting cancer should be cautious [61]. On the other hand, small molecule activators might be an attractive approach. However, several factors call for precautions in targeting PKM2. (1) PKM2 is also expressed in normal tissue [16, 17] and the function of PKM2 in normal tissues has not yet been determined; (2) genetic changes in PKM2 have not been reported in primary cancers; (3) despite modulation of PKM2 which affects formation of xenograft tumors, whether tissue-specific manipulation of PKM2 impacts tumorigenesis is still on the waiting list; (4) as PKM2 was detected in cancer stroma [113, 114], whether it plays a role in tumorigenesis by affecting cancer-associated fibroblasts is not clear; (5) while aerobic glycolysis has been a hot topic in the last decade, its impact on cancer stem cells (CSCs) has not been addressed. As it is becoming increasingly clear that CSCs play a critical role in tumorigenesis, especially in tumor progression and metastasis [115], it would appear critical to understand whether targeting cancer metabolism in general and PKM2 in particular will have an inhibitory effect on CSCs. This knowledge became important as it was suggested that glioma CSCs (GSCs) may not use aerobic glycolysis to the same degree as differentiated cancer cells. Thus, targeting PKM2 or cancer metabolism may still spare GSCs [116].
